# Supplementary material for: Optical Identification of Diabetic Retinopathy Using Hyperspectral Imaging
Source: J Pers Med. 2023 Jun 1;13(6):939. doi: 10.3390/jpm13060939 (PMC10303351; doi:10.3390/jpm13060939)
Supplement: Supplementary file 1 [file jpm-13-00939-s001.zip › jpm-2360504-supplementary.pdf]

Journal of Personalized Medicine

Article

Optical Identification of Diabetic Retinopathy using Hyperspectral Imaging: Supplementary File

Ching-Yu Wang [1], Arvind Mukundan [2], Yu-Sin Liu [2], Yu-Ming Tsao [2], Fen-Chi Lin [3,*], Wen-Shuang Fan [1] and Hsiang-Chen Wang [2,4,*]

1 Department of Ophthalmology, Dalin Tzu Chi Hospital, Buddhist Tzu Chi Medical Foundation, Chiayi 62247, Taiwan; s19001052@gmail.com (C.-Y.W.); wsfan@tzuchi.com.tw (W.-S.F.)
2 Department of Mechanical Engineering, National Chung Cheng University, Chiayi 62102, Taiwan; d09420003@ccu.edu.tw (A.M.); dan102030440@gmail.com (Y.-S.L.); d09420002@ccu.edu.tw (Y.-M.T.)
3 Department of Ophthalmology, Kaohsiung Armed Forces General Hospital, Kaohsiung 80284, Taiwan;
4 Director of Technology Development, Hitspectra Intelligent Technology Co., Ltd., Kaohsiung 80661, Taiwan
* Correspondence: eses.taiwan@gmail.com (F.-C.L.); hcwang@ccu.edu.tw (H.-C.W).

Abstract: This article provides the supplementary file for Hyperspectral Imaging Technique for Classifying Arteries and Veins in Fundus Images. Section 1 provides an overview of the principle of retinal fundus camera. Section 2 gives a brief description of the principle of retinal fluorescein angiography while section 3 talks on the principle of optical coherence tomography of the retina. Section 5 explain about the Gabor filter and the necessary equations used in this study.

Keywords: Hyperspectral Imaging; Fundus Imaging; Principal Component Analysis

Citation: Wang, C-Y.; Mukundan, A.; Liu, Y-S.; Tsao, Y-M.; Lin, F-C.; Fan, W-S.; Wang, H-C. Optical Identification of Diabetic Retinopathy using Hyperspectral Imaging. *J. Pers. Med.* **2023**, *13*, 939. https://doi.org/10.3390/jpm13060939

Academic Editor(s): Yousif Subhi

Received: 06 April 2023
Revised: 23 May 2023
Accepted: 30 May 2023
Published: 1 June 2023

Copyright: © 2023 by the authors. Licensee MDPI, Basel, Switzerland. This article is an open access article distributed under the terms and conditions of the Creative Commons Attribution (CC BY) license (https://creativecommons.org/licenses/by/4.0/).

1. The principle of retinal fundus camera

The fundus camera consists of imaging, lighting, and observation systems. To avoid the influence of other factors like reflected light on the cornea, the design of the imaging system included three parts: the eye objective lens, the imaging objective lens, and the negative film, which were different from the imaging mode of general cameras. The lighting system had two light sources, among which the first was used for fundus lighting when the camera was focused, and the second was the flash, used for increasing the brightness of fundus lighting upon shooting.

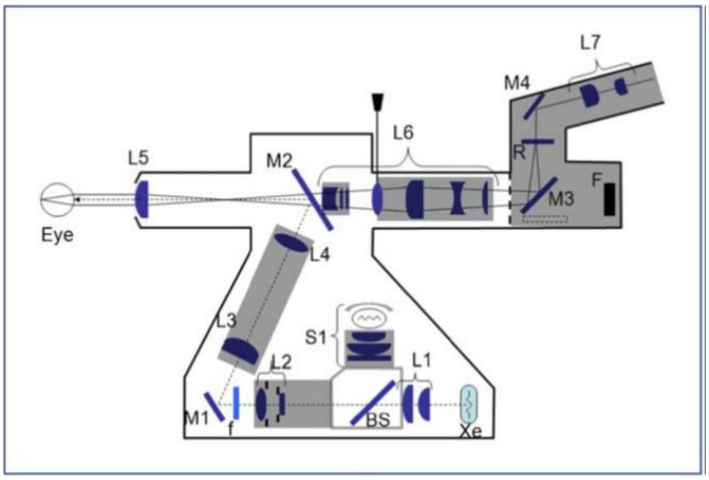

Figure S1. is a schematic diagram of the Zeiss fundus camera. Xe refers to the xenon flash; L1 refers to the concentrator; S1 refers to the light source of the observation system; L2-7 refers to the optical element of the imaging system; BS refers to the spectroscope; f refers to the filter; M1-4 refers to the

reflector; F refers to the negative film of the camera; R refers to the aiming target; the dotted line is the input path of the light source, and the solid line is the observation path [1].

2. Gabor filter

Gabor filter is a linear filter commonly used for edge detection. As shown in Figure S3, a two-dimensional Gabor filter is a Gaussian function modulated by sinusoidal plane waves. Given that its representation in the spatial domain and frequency domain is similar to human biological vision specificity, it can intuitively describe the structural information [3], such as the spatial position of images, directional selectivity, and spatial frequency (scale).

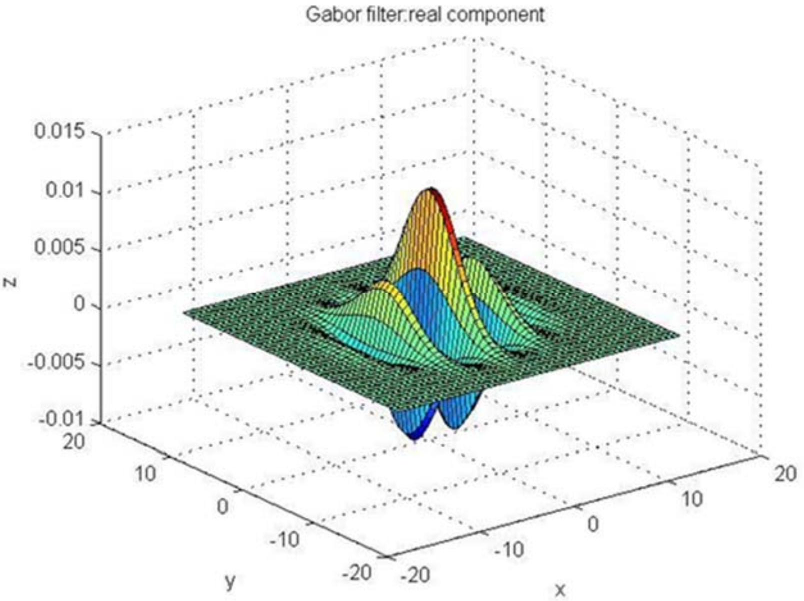

Figure S2. The Schematic Diagram of a Two-dimensional Gabor Filter**.**

The mathematical equation of the Gabor filter can be expressed as Eq. S1 and Eq. S2:

Real component:

$$\mathbf{g}(\mathbf{x},\mathbf{y}) = \frac{1}{2\pi\sigma_x\sigma_y}\mathbf{exp}\left[-\frac{1}{2}\left(\frac{x'^2}{\sigma_x^2}+\frac{y'^2}{\sigma_y^2}\right)\right]\boldsymbol{cos}(2\pi f_0 x') \quad \text{(S1)}$$

Imaginary component:

$$\mathbf{g}(\mathbf{x},\mathbf{y}) = \frac{1}{2\pi\sigma_x\sigma_y}\mathbf{exp}\left[-\frac{1}{2}\left(\frac{x'^2}{\sigma_x^2}+\frac{y'^2}{\sigma_y^2}\right)\right]\boldsymbol{sin}(2\pi f_0 x') \quad \text{(S2)}$$

In particular, $\mathbf{g}(\mathbf{x},\mathbf{y})$ is the response value of the image that is processed by the Gabor filter; $\boldsymbol{f_0}$ is the frequency of the curve; and $\boldsymbol{\sigma_x}$ and $\boldsymbol{\sigma_y}$ are the standard deviations in the x and y directions, respectively.

For the edge extraction of fundus images, a series of filter responses can be obtained from the results of all angles in the range of $\left[\frac{-\pi}{2},\frac{\pi}{2}\right]$ through Gabor filter. The results of the coordinate transformation formula at different angles of rotation are shown in Eq. S3 and Eq. S4:

$$\mathbf{x}' = \mathbf{x}\cos\boldsymbol{\theta} + \boldsymbol{y}\sin\boldsymbol{\theta} \quad \text{(S3)}$$

$$\mathbf{y}' = -\mathbf{x}\sin\boldsymbol{\theta} + \boldsymbol{y}\cos\boldsymbol{\theta} \quad \text{(S4)}$$

In this equation, $(\mathbf{x}', \mathbf{y}')$ are the corresponding coordinates after the rotation of θ values. Various filter responses $\boldsymbol{G^{\theta}}(\mathbf{x}, \mathbf{y})$ can be obtained using Gabor filters $\mathbf{g}(\mathbf{x}, \mathbf{y})$ with different **θ values** in fundus images. Then, the absolute values of the real and imaginary components of each angle are added, and the square root is taken, as shown in Eq. S5:

$$\boldsymbol{sqrt}(\boldsymbol{G_{Y}^{\theta}}^{2} + \boldsymbol{G_{i}^{\theta}}^{2}) = \boldsymbol{G^{\theta}} \qquad (S5)$$

In the equation, $\boldsymbol{G_{Y}^{\theta}}$ is the real component response, and $\boldsymbol{G_{i}^{\theta}}$ is the imaginary component response. In order to obtain the vascular position more easily, only the maximum response $\mathbf{R}(\mathbf{x}, \mathbf{y})$ is retained in each pixel point $(\mathbf{x}, \mathbf{y})$, as shown in Eq. S6:

$$\mathbf{R}(\mathrm{x}, \mathbf{y}) = \boldsymbol{Max}(\boldsymbol{G^{\theta}}(\mathrm{x}, \mathrm{y})), \boldsymbol{\theta} = [-\frac{\pi}{2} : \frac{\pi}{180} : \frac{\pi}{2}] \qquad (S6)$$

3. Equations used for evaluating the metrics.

Sensitivity represents the hit rate of the correct judgment, also known as true positive rate (TPR), which is defined in Eq. (Ss7):

$$\text{Sensitivity} = \frac{\textbf{The number of pixels judged as arteries/veins correctly}}{\textbf{The original number of pixels of } \frac{\textbf{arteries}}{\textbf{veins}} \textbf{ in the fundus images}} \qquad (S7)$$

Precision is also known as positive predictive value (PPV), whose values represent the proportions where the locations of arteries and veins are judged correctly. The computation method is shown in Eq. (S8):

$$\textbf{Precision} = \frac{\textbf{The number of pixels judged as arteries and veins correctly}}{\textbf{The number of all pixels judged as arteries and veins}} \qquad (S8)$$

F1-score is a harmonic mean, and the computation method is shown in Eq. (S9):

$$\textbf{F1} - \textbf{score} = \frac{\textbf{2*Sensitivity*Precision}}{\textbf{Sensitivity+Precision}} \qquad (S9)$$

4. Principal Component Analysis

Principal Component Analysis is a commonly used method for multivariate statistics. Multivariate data will undoubtedly provide rich information for research, but if each indicator is analyzed separately, it will often fail to fully utilize and lose a lot of usefulness. Therefore, the goal of PCA is to convert high-dimensional data to low-dimensional data to reduce the amount of computation under the premise that the loss of information in the data is small. In order to achieve the above results, the process of PCA is to find some projection directions that maximize the variance in these projection directions, and these projection directions are orthogonal to each other. The larger the variance in the process of finding new orthogonality, the greater the variance in the corresponding positive direction. The submission contains more information to explain the geometric implications of PCA. In addition, PCA belongs to unsupervised learning in machine learning. It does not need to give labeled training data in advance, but only needs to obtain data for subsequent statistical processing to obtain useful information such as features in the sample, thus making PCA more widely used.

The main job of PCA is to map n-dimensional features onto k-dimensions, which are also called principal components with orthogonal features. First, find a set of coordinate axes with the largest variance from the original data, and then find the second coordinate axis that has the largest variance in the plane orthogonal to the first axis. You can find the third coordinate axis and the first and second axes in order. In the orthogonal plane, the variance is the largest, and most of the variance can be included in the first k coordinate axes. Therefore, as a basis for ignoring the remaining coordinate axes, it is equivalent to reducing the original data to k dimension. In this study, the PCA algorithm is implemented based on the SVD decomposition of the covariance matrix. By calculating the covariance matrix of the data matrix and then obtaining the eigenvectors of the eigenvalues

of the covariance matrix, the eigenvectors corresponding to the k features with the largest eigenvalues are selected. A matrix is composed to achieve data dimensionality reduction. SVD decomposes the covariance matrix to implement the PCA algorithm. It is necessary to input the data set first, and then subtract the respective averages from each data and then calculate the covariance matrix (A). The most basic calculation is the covariance of two different values X and Y of the variance to explain the PCA principle used in this study. The two formulas are as follows:

$$\mathrm{var}(x) = \frac{\sum_{i=1}^{n}(x_i - \overline{x})^2}{n-1} \tag{S1}$$

$$\mathrm{cov(x, y)} = \frac{\sum_{i=1}^{n}(x_i - \overline{x})(y_i - \overline{y})}{n-1} \tag{S2}$$

Among them, the variance is used to calculate one-dimensional features, that is, for different sample values of the same feature, and the covariance must meet at least two-dimensional features and the variance is a special case of the covariance. In addition, the divisors of both are n-1 to satisfy the unbiased estimator, which can make the expected value of the sample variance better approximate the population variance. When the amount of variation is more than two-dimensional, a covariance matrix is needed to deal with the situation of multiple variables and calculate the variation between two values. In addition, the covariation matrix has symmetry, so for a matrix where n is data, only to calculate the Cn2/2 degree variance, this matrix can be listed as:

$$\begin{bmatrix} \mathrm{cov}(x,x) & \mathrm{cov}(x,y) & \mathrm{cov}(x,z) \\ \ldots & \mathrm{cov}(y,y) & \mathrm{cov}(y,z) \\ \ldots & \ldots & \mathrm{cov}(z,z) \end{bmatrix} \tag{S3}$$

The third step uses the SVD principle to calculate the eigenvalues and eigenvectors of the covariance matrix. The covariance matrix can be decomposed into the following forms:

$$\mathrm{A}=U\Sigma V^T \tag{S4}$$

The orthogonal vector in U is the left singular vector, and its composition is to find the eigenvalues and eigenvectors of AAT, and AAT is the divergence vector of A, and normalize the eigenvectors to obtain; if a vector v is the eigenvectors of matrix A, which can be expressed as:

$$Av = \lambda v \tag{S5}$$

Therefore, a matrix can obtain a set of eigenvectors. Similarly, the eigenvalues and eigenvectors of ATA are calculated and then normalized to obtain V, also known as the right singular vector. Σ can be obtained by taking the square root of the eigenvalues of AAT or ATA. Sort the eigenvalues of the left singular value matrix from large to small and take the largest k in the front, then use the k eigenvectors as column vectors to form an eigenvector matrix, and finally reduce the original data n eigenvectors to k in a new space constructed from a feature vector.

5. Binarization

In this study, Ridler and Calvard's iterative-based binarization method is used to find the threshold t of the image. Since the grayscale image is between 0 and 255, we need to choose a threshold that can cut the blood vessel area out, but also to reduce background and noise interference as much as possible. When the image to be processed consists of a

target with a large difference in gray value and the rest of the background, and if the grayscale distribution of the pixels in the target area is uniform, and the pixels in the background area are also uniformly distributed in another grayscale, you can select the corresponding gray value in the middle of these two peaks is used as the threshold value, and the iterative method is to obtain this result by repeating the fixed steps. First, we average the gray value of the overall image as the initial threshold, then use the initial threshold to divide the image into two groups, then calculate the average gray value of the two groups respectively and average them to get a new and then repeat the above steps to continuously generate a new threshold and take the difference between the previous thresholds. When the difference is less than a preset parameter, the final threshold is obtained. Ideally, this threshold will be the same as the original image peak middle valley value. When setting the threshold value in the fourth step, it is inevitable that there will be some small white spots due to uneven brightness or poor quality in the image, so it will be divided into the same area with the blood vessels, which will affect the final identification result. So, we can use the equation provided by the matlab program itself: bwareaopen to help filter out. This function can be used to set the connected objects with less than how many pixels to delete, and the pixel connectivity is initially present. As long as the pixel edges are in contact with each other, these pixels are connected, so for Programmatically, they are the same object, and the pixels are larger than the set value and can be deleted together

Author Contributions: Conceptualization, C.-Y.W., H.-C.W.; methodology, A.M.; Software, H.-C.W. and A.M.; validation, C.-Y.W., Y.-M.T. and H.-C.W.; formal analysis, C.-Y.W. and H.-C.W.; investigation, W.-S.F. and H.-C.W.; resources, Y.-M.T. and H.-C.W.; data curation, Y.-S.L., A.M., H.-C.W.; writing—original draft preparation, A.M.; writing—review and editing, A.M. and F.-C.L.; supervision, W.-S.F., F.-C.L. and H.-C.W.; project administration, W.-S.F., H.-C.W. All authors have read and agreed to the published version of the manuscript.

Funding: This research was supported by the Ministry of Science and Technology, the Republic of China under grants MOST 111-2221-E-194-007. This work was financially or partially supported by the Kaohsiung Armed Forces General Hospital research project MND-MAB-110-141 and Dalin Tzu Chi Hospital, Buddhist Tzu Chi Medical Foundation-National Chung Cheng University Joint Research Program DTCRD112-C-11 in Taiwan.

Institutional Review Board Statement: The study was conducted according to the guidelines of the Declaration of Helsinki, and approved by the Institutional Review Board of Dalin Tzu Chi General Hospital (B11201035).

Informed Consent Statement: Written informed consent was waived in this study because of the retrospective anonymized nature of study design.

Data Availability Statement: The data presented in this study are available in this article.

Conflicts of Interest: The authors declare no conflicts of interest.

References

1. Littmann, H. *Die Zeiss Funduskamera Ber. 59. Zusammenkunft Deutsch. Ophthalmolog. Gesellsch., Heidelberg 1955*; Verlag Bergmann: Munchen, Germany, 1995; p. 318.
2. Morgner, U.; Drexler, W.; Kärtner, F.X.; Li, X.D.; Pitris, C.; Ippen, E.P.; Fujimoto, J.G. Spectroscopic optical coherencetomography. *Opt. Lett.* **2000**, *25*, 111–113.
3. Siddalingaswamy, P.C.; Prabhu, K.G. Automatic detection of multiple oriented blood vessels in retinal images. *J. Biomed. Sci. Eng.* **2010**, *3*, 101–107.

Disclaimer/Publisher's Note: The statements, opinions and data contained in all publications are solely those of the individual author(s) and contributor(s) and not of MDPI and/or the editor(s). MDPI and/or the editor(s) disclaim responsibility for any injury to people or property resulting from any ideas, methods, instructions or products referred to in the content.
